# Case Report: Recurrence of Positive SARS-CoV-2 Results in Patients Recovered From COVID-19

**DOI:** 10.3389/fmed.2020.585485

**Published:** 2020-11-30

**Authors:** Ren-zi Zhang, Wang Deng, Jing He, Yu-yan Song, Chun-fang Qian, Qian Yu, Dao-xin Wang

**Affiliations:** ^1^Department of Respiratory and Critical Care Medicine, Second Affiliated Hospital of Chongqing Medical University, Chongqing, China; ^2^Chongqing Medical Research Center for Respiratory and Critical Care Medicine, Chongqing, China; ^3^Department of Intensive Care Unit, Chongqing Public Health Medical Center, Chongqing, China; ^4^Department of Tuberculosis, Chongqing Public Health Medical Center, Chongqing, China

**Keywords:** coronavirus disease 2019, severe acute respiratory syndrome coronavirus 2, IgM, IgG, nucleic acid detection

## Abstract

**Background:** Coronavirus disease 2019 (COVID-19) is spreading throughout the world. Limited data are available for recurrence of positive severe acute respiratory syndrome coronavirus 2 (SARS-CoV-2) results in patients with long duration of COVID-19.

**Methods:** We reported four cases recovered from COVID-19 with recurrence of positive SARS-CoV-2 results during the long-term follow-up.

**Results:** The four patients recovered from COVID-19 showed recurrence of positive SARS-CoV-2 results for more than 120 days with no symptoms and normal chest CT scan.

**Conclusions:** The dynamic surveillance of SARS-CoV-2 by nucleic acid detection and serological assays is important for asymptomatic patients who might be potentially infectious.

## Introduction

With the increasing number of patients recovered from Coronavirus disease 2019 (COVID-19), more attention should be paid to the follow-up of these patients. Here we reported four cases with recurrence of positive SARS-CoV-2 results in patients recovered from COVID-19 for more than 120 days.

## Case Presentation

### Case 1

**Figure 1:**
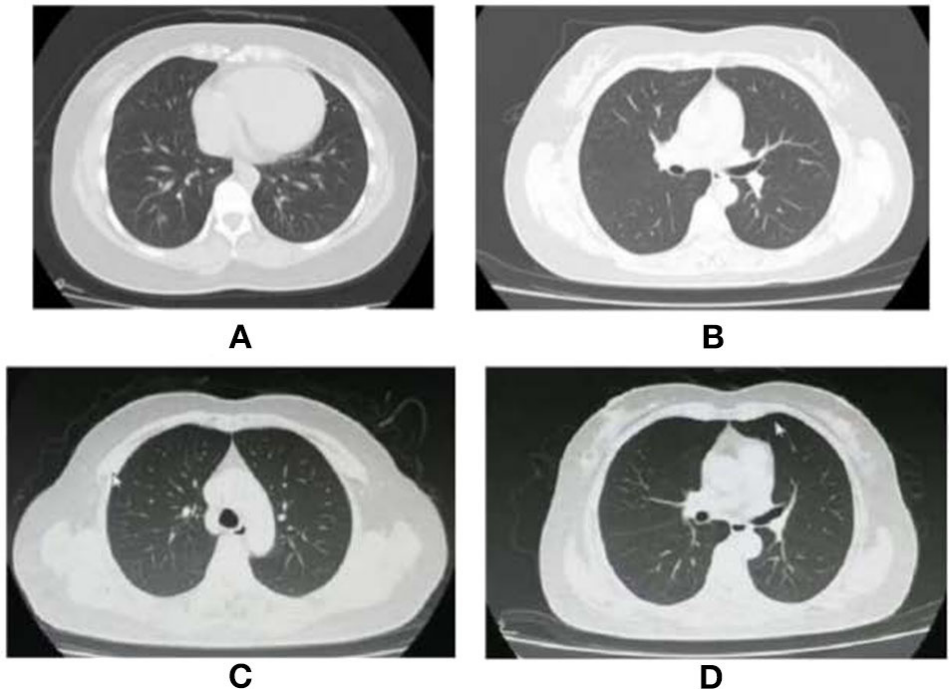
Chest CT images of two cases with COVID-19. **(A)** The Chest CT images of the Case 1 showed ground-glass opacities in basal segment of the lower lobe of the right lung on January 2. **(B)** The Chest CT images of the Case 1 showed almost normal on March 23. **(C)** The Chest CT images of the Case 2 showed almost normal on April 7. **(D)** The Chest CT images of the Case 2 showed almost normal on June 21.

A 36-year-old woman had a fever of 38.3°C with cough and shortness of breath on January 29, 2020. She lived with four family members, all of whom were diagnosed as COVID-19 on January 26. Real-time reverse-transcription polymerase chain reaction (qRT-PCR) assay of nasopharyngeal swab-obtained materials showed a positive result of SARS-CoV-2. The chest computed tomography (CT) scan showed ground-glass opacities in basal segment of the lower lobe of right lung (Figure 1A). The patient was admitted to Chongqing Public Health Medical Center. She was treated with lopinavir/ritonavir (200/50 mg, 2 tablets, biw) from January 30 to February 20, hydroxychloroquine (400 mg, biw on the first day, and 200 mg, biw on the following day) from February 20 to February 25, atomized inhalation of interferon α-2b (500,000 U, biw) from January 30 to March 1, and thymalfasin (1.6 mg, biw) from March 7 to March 9, supplemented with Chinese Medicinal therapy. The two nasopharyngeal swabs collected on March 8 and 9 were both negative for SARS-CoV-2 by RT-PCR tests. On March 9, the patient was discharged and went to a designated hospital for quarantine and observation. On March 23, the nasopharyngeal swab result of SARS-CoV-2 by RT-PCR test was positive again. She was readmitted to Chongqing Public Health Medical Center. The chest CT scan showed no abnormality (Figure 1B). The laboratory data contained no abnormalities. The patient was treated with thymalfasin (1.6 mg, biw) for April 17 to May 17 and hydroxychloroquine (400 mg, biw on the first day, and 200 mg, biw on the following day) for April 18 to April 27 in combination with Chinese herbs. Serum immunoglobulin M (IgM) and immunoglobulin G (IgG) specifically for SARS-CoV-2 antigens were detected on May 21 by using magnetic chemiluminescence enzyme immunoassay kits (Bioscience Biotechnology Co.) (Table 1). The treatment was switched to atomized inhalation of interferon α-2b (500,000 U, biw) from May 21 to July 2. On July 2, the patient was discharged and maintained home quarantine after 14 consecutively negative results of SARS-CoV-2 by nasopharyngeal swab tests.

**Table 1:** The IgG and IgM titers of four patients.

	**Case 1**			**Case 2**	
**Date**	**IgG**	**IgM**	**Date**	**IgG**	**IgM**
5–21	12.515	1.573	5–21	22.614	2.682
5–24	10.805	1.512	5–24	20.311	2.451
5-−26	11.922	1.385	5–30	18.992	1.771
5–30	10.873	1.525	6–1	22.814	2.034
6–6	11.081	1.337	6–6	11.918	1.865
6-11	9.921	1.328	6–7	8.781	1.046
6-14	7.237	0.017	6–8	0.313	0.037
	**Case 3**			**Case4**	
**Date**	**IgG**	**IgM**	**Date**	**IgG**	**IgM**
3–27	9.517	0.122	5–26	6.593	2.168
4–3	5.023	0.097	6–3	5.916	2.635
4–6	5.927	0.092	6–6	5.382	2.323
4–14	4.645	0.066	6–15	3.907	1.367
4–18	5.238	0.107	6–19	4.203	1.238
4–24	4.615	0.113	6–22	3.447	0.231
4–27	4.513	0.089			
5–1	5.367	0.409			
5–9	4.425	0.225			
5–15	3.988	0.279			

*The normal range for IgG and IgM detection was 0–1*.

### Case 2

A 54-year-old woman had a fever, sore throat, and cough on February 14. She was diagnosed as COVID-19 by positive nucleic acid test for SARS-CoV-2 with a contact history of patients with fever in Wuhan, China. The chest CT showed multiple patchy shadows in the lower lobes of both lungs. The patient was treated with arbidol, lopinavir/ritonavir, and Chinese Medicine from February 16 to March 14 in Wuhan. Two consecutive results of nasopharyngeal swabs were negative. On March 14, the patient was discharged from hospital. During the 14-day isolation period, the patient had no symptoms and multiple nucleic acid tests were negative. She was required to be quarantined in a government-designated hospital for 14 days. She was transferred to Chongqing Public Health Medical Center due to the positive result of SARS-CoV-2 on April 7. The chest CT scan was almost normal (Figure 1C). The patient was treated with arbidol (200 g, biw) from April 7 to April 17, hydroxychloroquine (400 mg, biw on the first day, and 200 mg, biw on the following day) from April 16 to April 24, and thymalfasin (1.6 mg, biw) from April 16 to April 26, combined with Chinese herbs. However, the IgG and IgM against SARS-CoV-2 was positive on May 21 (Table 1). The treatment was changed to atomized inhalation of interferon α-2b (500,000 U, biw) from May 21 to June 21. On June 21, the chest CT scan was almost normal (Figure 1D). The patient was discharged and maintained home quarantine after 14 consecutively negative results of SARS-CoV-2 by nasopharyngeal swab tests.

### Case 3

A 50-year-old woman had fever and cough with a contact history of residents from Wuhan presenting the similar symptoms. She was confirmed as COVID-19 by positive nasopharyngeal swab of SARS-CoV-2 and was admitted to a local hospital on February 19. The patient had a history of chronic viral hepatitis B for more than 10 years. The chest CT showed multiple patchy ground-glass opacities in bilateral subpleural areas. The laboratory data of hepatitis B virus load was low. Other laboratory parameters were normal. The patient was treated with arbidol (200 g, biw) from February 22 to March 2, hydroxychloroquine (400 mg, biw on the first day, and 200 mg, biw on the following day) from February 24 to March 3, and lopinavir/ritonavir (200/50 mg, 2 tablets, biw) from March 10 to March 20, combined with Chinese Medicinal therapy. On March 22, she was discharged and went to a designated hospital. On March 27, the patient was transferred to Chongqing Public Health Medical Center for further treatment due to the positive nucleic acid test for SARS-CoV-2.The CT scan was almost normal. The IgG for SARS-CoV-2 was positive (Table 1). The patient was treated with thymalfasin (1.6 mg, biw) from April 6 to May 9, atomized inhalation of interferon α-2b (500,000 U, biw) from April 20 to May 9 and from May 15 to July 3, and hydroxychloroquine (400 mg, biw on the first day, and 200 mg, biw on the following day) from April 21 to April 23, combined with Chinese herbs. On July 3, the patient was discharged and maintained home quarantine after 14 consecutively negative results of SARS-CoV-2 by nasopharyngeal swab tests.

### Case 4

A 55-year-old man had fever of 37.5°C with headache and muscle soreness on February 3. He had a contact history of a confirmed COVID-19 case in the past 2 weeks. He was confirmed as COVID-19 by positive nucleic acid test. On admission, the chest CT images presented stripe shadows and focal ground-glass opacities in the upper and lower lobes of the left lung and middle and lower lobes of the right lung. The patient was treated with lopinavir/ritonavir, thymopentin, and Chinese Medicine. The three times of nasopharyngeal swabs collected on February 27, 28, and 29 were all negative for SARS-CoV-2, respectively. The anal swab test for SARS-CoV-2 by RT-PCR was negative on March 2. The patient was discharged on March 3. The patient was admitted to a government-designated hospital for centralized isolation after a 1-week home quarantine. On March 17, the nucleic acid test for SARS-CoV-2 was positive again. During the hospitalization, the nasopharyngeal swab tests for SARS-CoV-2 were two negative results. He went for medical observation for 2 weeks. On April 7, the nasopharyngeal swab test for SARS-CoV-2 was positive again. He was transferred to Chongqing Public Health Medical Center. The chest CT images presented stripe shadows and diffuse consolidation in both lungs. The patient was treated with thymalfasin (1.6 mg, biw) from April 7 to May 11 and hydroxychloroquine (400 mg, biw on the first day, and 200 mg, biw on the following day) from April 16 to April 24, combined with Chinese herbs. However, the IgG and IgM against SARS-CoV-2 were positive on May 26 (Table 1). The treatment was changed to atomized inhalation of interferon α-2b (500,000 U, biw) from May 26 to July 10. On July 24, the patient was discharged and maintained home quarantine after 14 consecutively negative results of SARS-CoV-2 by nasopharyngeal swab tests.

## Results

**Figure 2:**
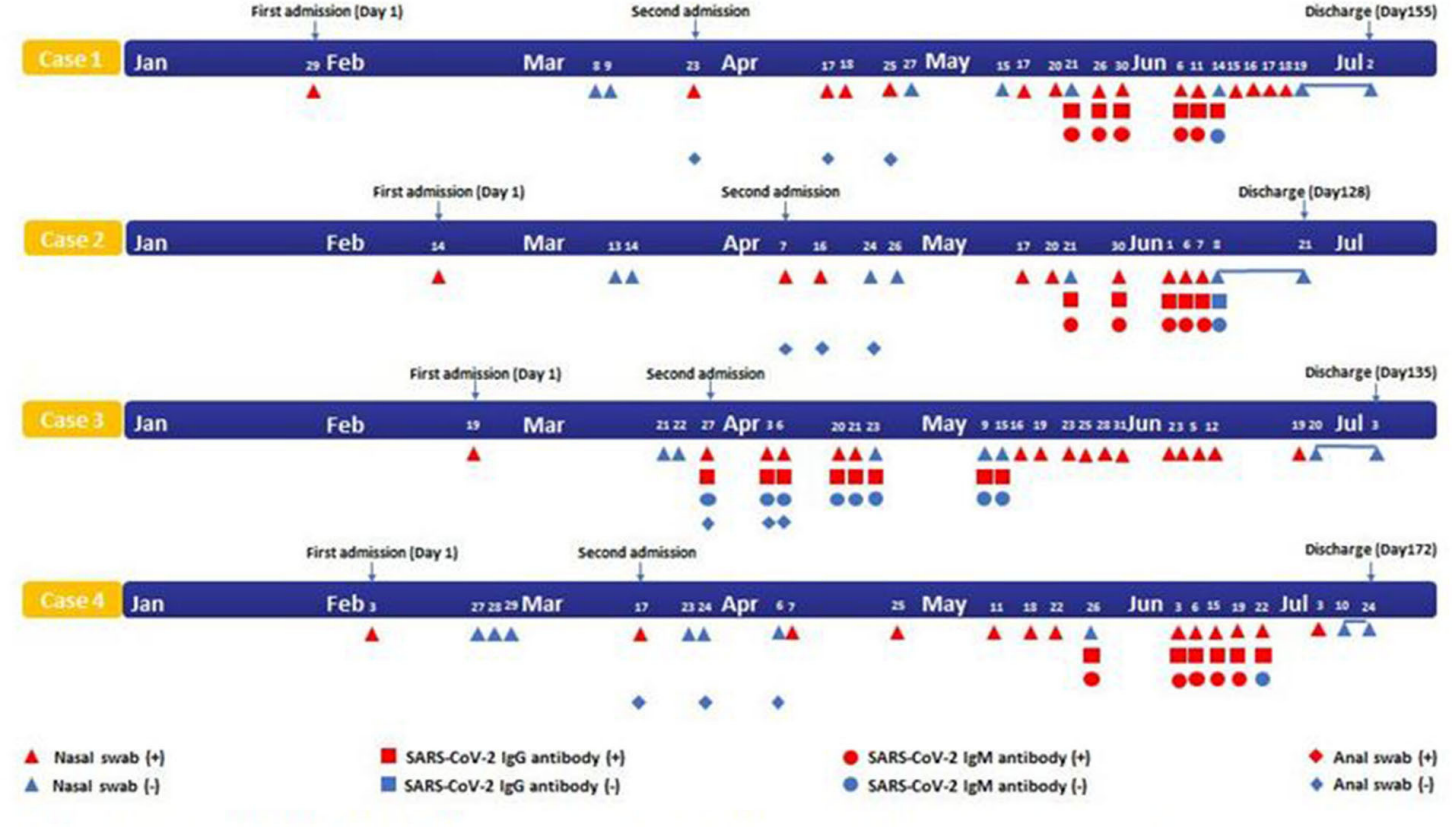
Timeline of four cases with SARS-CoV-2 infection. SARS-CoV-2, Severe acute respiratory syndrome coronavirus 2.

Four cases were confirmed as COVID-19 on the first admission. None had underlying diseases such as diabetes, hypertension, and cardiovascular disease. After treatment, they met the discharge standard for two consecutively negative nucleic acid tests for SARS-CoV-2 and then were isolated in a government-designated hospital for at least 14 days. However, they had positive RT-PCR results of SARS-CoV-2 with no symptoms after discharge (Figure 2). On the second admission, the chest CT scan of three cases was normal. The serological assays of these three cases were positive for IgG and IgM. Only one case was positive for IgG (Figure 2). Multiple antiviral treatments were used in combination with Chinese Medical therapy. Four cases were treated with psychotherapy for anxiety disorders. The disease duration of four patients were 128, 135, 155, and 172 days, respectively, after discharge. All the four patients did not have additional recurrences during the follow-up visit with negative results of SARS-CoV-2 by nasopharyngeal swabs tests after discharge from July to September.

## Discussion

In this report, all four patients met the discharge standard with two consecutively negative nucleic acid tests on the first admission. However, the nasopharyngeal swab test of SARS-CoV-2 had recurrence of positive results during the follow-up period. There was little possibility for reinfection due to no contact with other patients with COVID-19 during the quarantine. The course of patients with COVID-19 for more than 90 days has been reported (1). Unexpectedly, the disease duration of the four patients lasted for more than 120 days, suggesting the longest duration reported outside Wuhan, China.

The nasopharyngeal swab test of SARS-CoV-2 by RT-PCR is currently the most common method for diagnosis of COVID-19. With the progression of SARS-CoV-2 infection, the virus could migrate from the upper respiratory tract to the lower respiratory tract and lungs (2, 3), resulting in insufficient viral load in the upper respiratory tract, which may explain the negative result of nasopharyngeal swab test (4). Currently, limited methods are available to determine the viral load and activity. Additionally, lack of experience with sample collection, transportation, and inspection could also lead to the false-negative results (5). Therefore, the detection rate and sensitivity could be improved by multisite specimen collection including alveolar lavage fluid, sputum, and serological assays (6). Furthermore, the nucleic acid test should be performed repeatedly for highly suspected patients to avoid misdiagnosis. The quarantine period should be prolonged for at least 50 days for recovered patients in order to avoid virus carrier transmission and identify patients that may pose a risk for the future outbreaks (7).

All four patients were discharged after meeting the discharge criteria on the first admission, indicating that the treatment was effective. The antiviral treatment temporarily cleared the virus, resulting in the negative result of SARS-CoV-2. However, it could still take days for the immune system to completely eliminate the remaining virus in the body (8), leading to intermittent release of the virus for recurrence of positive results. Once the virus had replication and intermittent shedding, the RT-PCR results reverted to positive in the discharged patients (7). Continuous antiviral therapy should be considered especially for the patients with repeatedly positive results of SARS-CoV-2 (9). The asymptomatic patients had a significantly longer duration of viral shedding than the symptomatic patients as previously reported (10). There still lacked the evidence for the viral clearance or the duration of viral shedding after initial infection. The “dead” virus or viral fragments without replication may also contribute to repeatedly positive results without potential infectiousness (4, 11).

All four patients were diagnosed as COVID-19 on the first admission. Case 3 had chronic hepatitis B virus for more than 10 years. Whether hepatitis B virus and SARS-CoV-2 affected each other still remains unclear. The nasopharyngeal swab tests of all four patients were recurrently positive on the second admission with negative results of anal swab tests, indicating the less possibility of oral-fecal transmission. The family members of Case 1 and Case 2 did not have recurrence of positive results of SARS-CoV-2 after discharge. Only four severe patients in total of 576 patients with COVID-19 were recurrently positive by nasopharyngeal swab tests in Chongqing, China. The four patients were isolated with the average duration of positive RT-PCR test for <40 days. The severe patients with COVID-19 may have a faster conversion from positive to negative by RT-PCR test.

The four patients were asymptomatic with normal chest CT scan and laboratory data on the second admission. The nasopharyngeal swab tests of SARS-COV-2 were recurrently positive. Case 1, Case 2, and Case 4 were also continuously positive for IgG and IgM. The weak production of the virus-specific IgG and IgM could lead to asymptomatic or mild patients which could be a long-term carrier transmission (12). Asymptomatic patients had lower IgG titers and shorter duration due to a reduced immune response (10), which suggested that likelihood of “herd immunity” was low. After recovery from COVID-19, prevention of reinfection by a protective antibody and the duration of antibody protection still need to be determined. Therefore, the use of “immunity passports” for COVID-19 could be risky. We speculate that the presence of IgG does not have a long-term protective effect in some situations.

Serological assays in combination with viral nucleic acid test should be performed to screen for asymptomatic or suspected patients in order to reducing the false-negativity by RT-PCR test alone (13). The dynamic surveillance of SARS-CoV-2 by RT-PCR test combined with serological assays should be of significant value for viral infectiousness. These tests will also be informatively important for the diagnosis and prognosis of COVID-19.

## Data Availability Statement

The original contributions presented in the study are included in the article/supplementary materials, further inquiries can be directed to the corresponding author/s.

## Ethics Statement

Written informed consent was obtained from the individual(s) for the publication of any potentially identifiable images or data included in this article.

## Author Contributions

RZ, WD, and DW had full access to all of the data in the study and take responsibility for the integrity of the data and the accuracy of the data analysis, designed the study, and wrote the paper. JH, CQ, and QY were responsible for the acquisition, analysis, or interpretation of the data. DW contributed to the revision of the manuscript for important intellectual content. All authors have read and approved the manuscript.

## Conflict of Interest

The authors declare that the research was conducted in the absence of any commercial or financial relationships that could be construed as a potential conflict of interest.
